# Epitaxial SiGeSn Alloys for CMOS-Compatible Thermoelectric Devices

**DOI:** 10.1021/acsaem.5c00733

**Published:** 2025-06-18

**Authors:** Patrizio Graziosi, Damiano Marian, Andrea Tomadin, Stefano Roddaro, Omar Concepción, Jhonny Tiscareño-Ramírez, Prateek Kaul, Agnieszka Anna Corley-Wiciak, Dan Buca, Giovanni Capellini, Michele Virgilio

**Affiliations:** † 204549 CNR - ISMN, Via P. Gobetti 101, Bologna 40129, Italy; ‡ Dipartimento di Fisica, 9310 Università di Pisa, Largo Bruno Pontecorvo 3, Pisa 56127, Italy; § NEST, CNR Istituto Nanoscienze, piazza San Silvestro 12, Pisa 56127, Italy; ∥ Peter Gruenberg Institute 9 (PGI-9) and JARA-Fundamentals of Future Information Technologies, Forschungszentrum Juelich, Juelich 52428, Germany; ⊥ IHP - Leibniz Institute for High Performance Microelectronics, Frankfurt (Oder) 15236, Germany; # European Synchrotron Radiation Facility, 71 avenue des Martyrs, CS 40220, Grenoble 38043 Cedex 9, France; ∇ Dipartimento di Scienze, Università degli Studi Roma Tre, Viale G. Marconi 446, Roma 00146, Italy

**Keywords:** SiGeSn, thermoelectrics, CMOS, Boltzmann transport, lattice thermal properties

## Abstract

The integration of thermoelectric devices into mainstream microelectronic technological platforms could be a major breakthrough in various fields within the *so-called* Green-IT realm. In this article, the thermoelectric properties of heteroepitaxial SiGeSn alloys, an emergent CMOS-compatible material system, are evaluated to assess their possible application in thermoelectric devices. To this purpose, starting from the experimentally low lattice thermal conductivity of SiGeSn/Ge/Si layers of about ∼1 to 2 W/m·K assessed by means of 3-ω measurements, the figure of merits are calculated through the use of Boltzmann transport equation, taking into account the relevant intervalley scattering processes, peculiar of this multivalley material system. Values for the figure of merit *ZT* exceeding 1 have been obtained for both p- and n-type material at operating temperatures within the 300–400 K range, i.e., at typical on-chip temperatures. In this interval, the predicted power factor also features very competitive values on the order of 20 μW/cm ·K^2^. Our finding indicates that this emergent class of Si-based materials has extremely good prospects for real-world applications and can further stimulate scientific investigation in this ambit.

## Graphical abstract

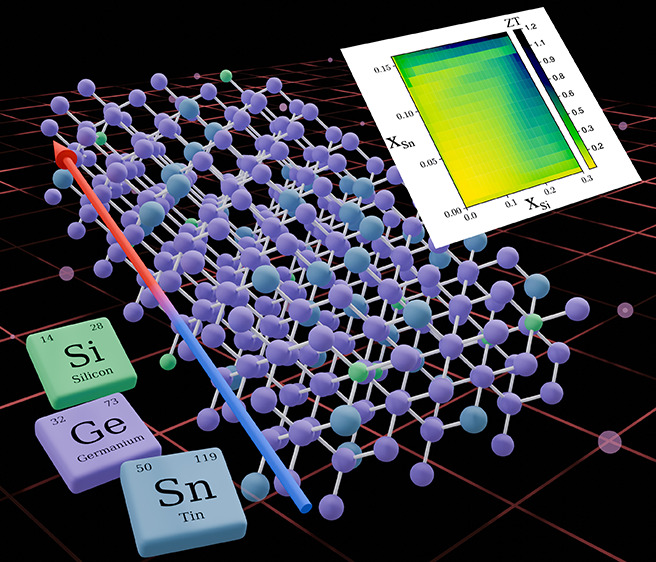

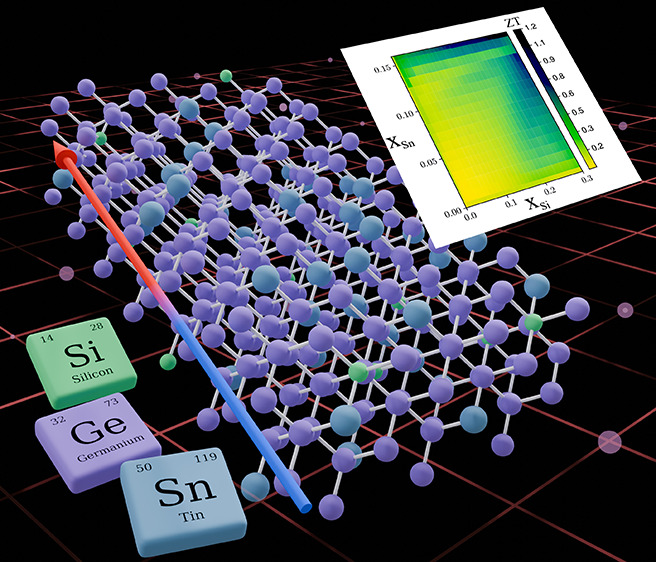

## Introduction

Thermoelectric (TE) devices provide a solution for direct energy conversion for applications and technologies that require a reliable source, rather than an efficient one.^1^ Thermoelectric generators (TEG) are used to convert low-grade heat into energy to power systems and devices for a variety of applications,^2^ e.g., wearable health monitoring,^3^ automotive^4^ and aerospace technologies,^5^ and manufacturing.^6^ Despite these achievements, the diffusion of TEG remains limited in large consumer markets due to major drawbacks of TE technologies available to date:^7^ (i) low conversion efficiency -especially for room temperature operations-; (ii) challenges in TEG miniaturization and integration in mass production manufacturing, leading to relatively high cost per device; (iii) TEG generator operating at room temperature (RT) are usually based on toxic materials or materials of limited availability.^8^

The potential performance of a TE material is usually quantified by the dimensionless figure of merit^9^ $ZT=\frac{S^{2}\sigma T}{\kappa }$ , where *S* is the Seebeck coefficient and σ and κ are the electrical and thermal conductivity, respectively. A 300 K value of *ZT* ≈ 1 is required for realizing a device with an efficiency of practical use (about 15% of the Carnot limit). An ideal TE material should then feature a low κ, needed to preserve the *T* gradient across the device while maintaining excellent electrical transport properties. However, there are well-known constraints to the optimization of the material parameters. As a matter of fact, σ in semiconductors can be increased by leveraging on the doping concentration, but as dictated by the Wiedemann–Franz law, this also induces larger κ values, due to the simultaneous increase of the electronic contribution κ_e_ to the thermal conductivity, thus imposing a trade-off to the optimal carrier density.^10^

Si, Ge, and their alloys are CMOS-compatible materials and have been used in high temperature TEG devices having a high *ZT* value peak at around 1200 K, see Figure 1a. However, their ZT dramatically drops at 300 K, also in nanostructured devices, engineered in the past decades in an attempt to enhance the TE performance of the bulk material, leveraging the modification of both the charge and heat transport properties of multilayer or grained material, as discussed in refs 11–13 . For this reason, today SiGe alloys are employed only at elevated temperatures in niche applications, as, e.g., for radioisotope thermoelectric generators in space missions. On the other hand, commonly used TE materials, such as Bi_2_Te_3_ and PbTe, exhibit high *ZT* values at room temperature (Figure 1a) due to their low thermal conductivity, but their toxicity and incompatibility with silicon-based microelectronics standards prevent their widespread use in real-world applications.^14^ Consequently, in view of potential large-scale applications integrated with consumer electronics, TE materials should be based on an innovative, CMOS-compatible, and nontoxic material system, having thermal conductivity at 300 K suppressed by efficient phonon–phonon scattering processes but still characterized by high charge carrier conductivity.

**Figure 1:**
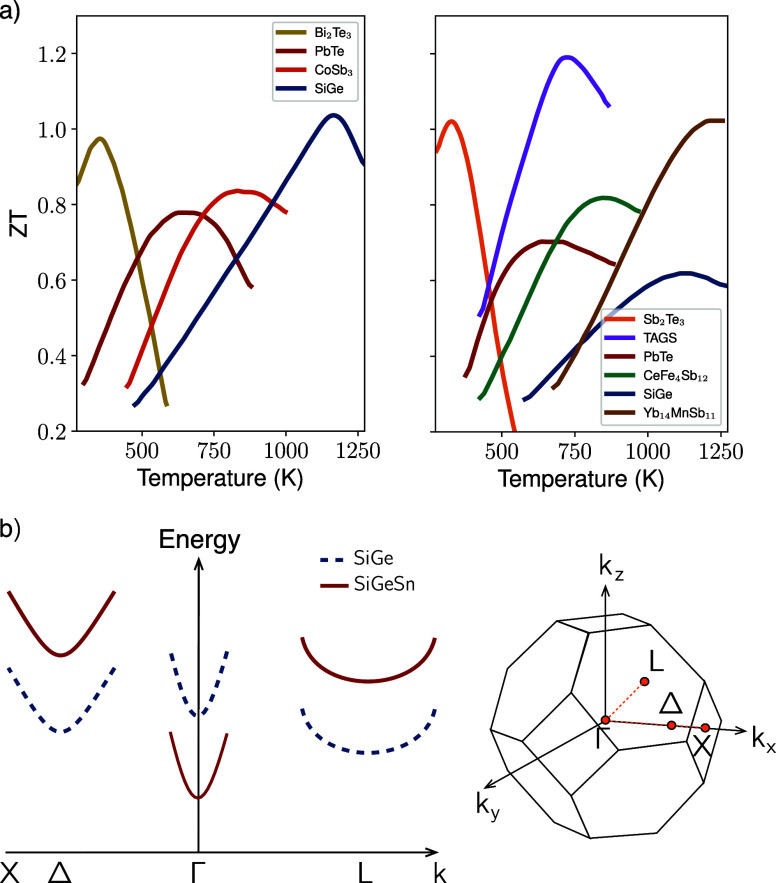
(a) *ZT* figure of merit as a function of temperature for different semiconductor n-type (left) and p-type (right) materials. Data taken from ref 16. (b) Left: schematic conduction band structure of a Ge-rich SiGe alloy (blue dashed). Alloying with Sn induces the *relative* downshift of the Γ_c_ band edge (solid curve) with respect to the Δ and L ones. L, Γ_c_, and Δ points conduction minima are typically found within a 100–200 meV energy range. Right: first Brillouin zone showing the position of these high symmetry points.

Ge-rich SiGeSn ternary alloys have recently emerged as a transformative Group IV material system. As a matter of fact, the addition of Sn into the SiGe matrix introduces significant changes in the material’s electronic and thermal properties since Sn incorporation: (i) dramatically reduces the lattice thermal conductivity due to the high mass contrast with the Si and Ge ions; (ii) can induce a down-shift of the Γ conduction valley below the energy of the L-point minimum, thus realizing a direct bandgap semiconductor material as schematically depicted in Figure 1b. Consequently, in n-type systems, larger electron mobilities with respect to those of SiGe can be obtained. The rationale for this mobility enhancement is related to the proximity of the Δ-, L-, and Γ-point band edges, typically lying within a ∼100 meV range in this multivalley quasi-direct semiconductor. It follows that alloying with Sn induces a significant increase of the carrier population in the Γ-valley, which features a lighter transport mass with respect to the L and Δ ones.^15^ Furthermore, the nonpolar character of SiGeSn alloys contributes to reducing the thermally induced suppression of the electrical conductivity, due to the absence of Fröhlich interaction.

The Si_*x*_Ge_1–*x*–*y*_Sn_*y*_ material system has come under the spotlight thanks to the recent advancements in the epitaxial growth of high-quality Ge-rich SiGeSn layers on Si-based Ge/Si virtual substrates, using tools and processes compatible with the standards of the Si-CMOS microelectronics industry.^6^ In particular, the possibility of extensive band gap engineering through composition has attracted the silicon photonics community.^17^ As a matter of fact, the so-called “directness” in Ge-rich alloys, obtained by Sn incorporation as stated above, has a huge positive impact on the radiative recombination efficiency of the material, leading to the demonstration of an optically pumped GeSn-based laser operating at room temperature^18^ or a cw-electrically pumped laser based on SiGeSn/GeSn multi quantum wells.^19^ The directness is also exploited in advanced microelectronic devices such as tunnel-FET, where the direct band gap increases the device performances thanks to more efficient tunneling rates of the Γ-valley electrons, which, for momentum conservation reasons, do not require phonon assistance.^20^ Finally, since the optimization of *ZT* usually requires high carrier densities, it is important to notice that, thanks to the aforementioned recent advances, doping of Ge-rich SiGeSn layers with active impurity concentrations exceeding 10^20^ cm^–3^ has been achieved for both p-type and n-type systems.^21^

It is clear that the unequivocal demonstration of high SiGeSn TE performances around 300 K could pave the way to a multifunctional and integrated platform comprising electronic, photonic, and TE devices, manufactured within the Si-CMOS standard, with all the inherent advantages granted by the microelectronic technology. This multifunctional toolbox would greatly contribute to the *so-called* green-IT objectives. In fact, due to its scalability, environmental friendliness, and low cost, it can be employed in large-scale devices designed for energy-harvesting on-chip^22^ or chip cooling,^23^ temperature-tunable photonic circuits for optical computation, sensing, and lab-on-chip architectures.

In this context, given the very promising low lattice thermal conductivity κ_l_ observed in the past few years for GeSn,^24–26^ studies have emerged in the literature targeting the numerical prediction of κ_l_ also in Si_*x*_Ge_1–*x*–*y*_Sn_*y*_ alloys, in the entire^27^ or for a selected subset^28^ of the (*x*, *y*) parameter space. These findings are very promising for TE applications since they suggest a very robust suppression of κ_l_ with respect to SiGe and GeSn, with values as low as 1 W/m·K. As a matter of fact, promising TE properties at room temperature for n- and p-type SiGeSn polycrystalline have been reported in ref 29. Here, we move a step further in this direction by combining lattice thermal conductivity measurements with a comprehensive numerical investigation of the electronic transport properties of the Ge-rich Si_*x*_Ge_1–*x*–*y*_Sn_*y*_ heteroepitaxial alloys. In this way, we are able to assess the TE properties of the ternary material system at 300 and 400 K, in the entire portion of the (*x*, *y*) parameter region experimentally accessible, thus extending to the ternary SiGeSn system the investigation of GeSn, previously reported in ref 26 by some of the Authors. Indeed, owing to the very low solid solubility of Sn in both Si and Ge, only tin content below ≈0.2 can be obtained without compromising on the electrical transport properties.^30,31^

We first performed 3-ω experiments to measure the lattice conductivity of a Ge-rich Si_*x*_Ge_1–*x*–*y*_Sn_*y*_ sample set featuring different Sn concentrations. Subsequently, upon combining these data with literature values of κ_l_, we achieved an estimation of the lattice thermal conductivity across the entire (*x*, *y*) parameter region. As for the electronic transport properties and their contribution to the thermal conductivity, we relied on the Boltzmann transport equation, beyond the constant time relaxation approximation, as implemented in the ElecTra simulation suite,^32,33^ where particular care has been devoted to include all the intervalley scattering processes, peculiar of this multivalley material system, as well as bipolar effects which are active at around 300 K due to the relatively small value of the band gap. After validating the ElecTra results against experimental mobility data for GeSn, we systematically explored the alloy parameter space of the ternary material to optimize its *ZT* values at different temperatures.

Our findings suggest that p- and n-type SiGeSn alloys can achieve *ZT* values greater than 1 in the 300–400 K temperature range, a performance comparable to commercial TE materials. This significant result demonstrates the potential of SiGeSn to bridge the gap between high-temperature TE materials and room-temperature applications, offering a sustainable, nontoxic, and CMOS-compatible solution for next-generation thermoelectric devices.

## Methods and Validation

### Electronic Transport Properties

To estimate the figure of merit *ZT* and the power factor PF for p- and n-type Si_*x*_Ge_1–*x*–*y*_Sn_*y*_ alloys, we assessed along the [100] crystallographic direction their charge transport properties and the electronic contribution to the thermal conductivity κ_e_ by means of numerical simulations based on the Boltzmann transport equation, which allowed us to explore the parameter space spanned by the Si and Sn concentration, the doping density, and the lattice temperature. Due to the quasi-direct character of the SiGeSn material system, we have taken into account the carrier population in the different conduction valleys, which correspond to the minima at the Γ_c_, L, and Δ points of the Brillouin zone, as well as their interaction through intervalley scattering events. For this purpose, we relied on the ElecTra code,^32,33^ a well-established simulation suite, developed by one of the Authors, which in the past few years has been extensively tested with group IV elemental materials and SiGe alloys.

ElecTra offers the functionality of charge transport calculation beyond the constant relaxation time approximation by considering (i) the full energy/momentum/band dependence of the scattering rates, (ii) the distinction between intra- and interband transitions, and (iii) the bipolar transport resulting from the joint contribution stemming from the valence and conduction carriers. Moreover, to increase the accuracy, calculations are performed in a full-band approach using anisotropic scattering rates. The scattering mechanisms taken into account are the electron–phonon coupling with the acoustic and optical branches, the Coulomb interaction induced by charged donor/acceptor ions, and the alloy disorder potential.

Due to the ternary and multivalley character of the investigated material system, particular care has been devoted to upgrading the description of the alloy scattering since literature models deal mainly with single valley and/or III–V semiconductors.

To this aim, we assume random mixing without explicitly considering possible short-order and/or clustering effects.^34,35^ As a first step, the square modulus of the alloy scattering matrix element for Si_*x*_Ge_1–*x*–*y*_Sn_*y*_ has been linearly decomposed, as outlined in ref 36, in terms of the binary SiGe and GeSn ones, weighted according to the Si:Sn ratio in the ternary material. Moreover, as outlined in ref 37, for the SiGe case, to evaluate each binary scattering matrix element, we have distinguished between intra- and intervalley events, adopting specific coupling potentials whose values depend on the initial and final valley state. The alloy scattering potentials used in our calculations for SiGe are taken from the theoretical estimations obtained by means of tight-binding supercell calculations in ref 37, where a validation procedure is also discussed. Indeed, the authors of ref 37. demonstrate that using their alloy scattering parameters, the experimental mobility of SiGe is successfully reproduced in the entire compositional range. For the case of GeSn, we relied on the theoretical values resulting from the supercell simulations based on the density functional theory reported in ref 15. However, after the model calibration procedure discussed hereafter, a rescaling factor was applied to the set of GeSn alloy potentials for the conduction band. As for the electron–phonon coupling, the adopted phonon energies and scattering parameters are reported and discussed in the SI, where we also list the values for all of the other material parameters used in our simulations.

The aforementioned model calibration procedure was performed using a set of high-quality p- and n-type GeSn layers, featuring a thickness between 250 and 300 nm and different Sn concentrations spanning the 5–15% interval. These samples were grown on 200 mm Si(100) wafers by using an industry-compatible reduced-pressure chemical vapor deposition reactor. In order to enhance the crystal quality, a 300 nm thick Ge buffer layer was introduced to accommodate the significant lattice mismatch between Sn and Si.

N-type epilayers have been in situ doped by codeposition of Phosphorus, achieving carrier densities ranging in the 2.4–3.8 × 10^19^ cm^–3^, as probed by means of Hall measurements at 300 K. From Hall experiments, performed on nominally intrinsic samples, we also asses p-type carrier densities the varying in the 10^16^–10^18^ cm^–3^ range, depending on the Sn content. Details on the epitaxy and structural characterization of the samples can be found in refs 25 and 26. Carrier mobilities are shown in Figure 2a as functions of the Sn concentration. In the p-type material, mobility vs Sn content does not exhibit a clear trend. As a matter of fact, in this case, the scattered values of μ simply reflect the differences in the doping concentrations, with a peak value around 1100 cm^2^/V · s, obtained for the Ge_90_Sn_10_ sample, thanks to the lowest carrier density (1.2 × 10^16^ cm^–3^). Conversely, in the n-type sample set, we observe for Sn concentration in the 7–14% interval a well-defined monotonic increase of μ, from ∼230 to 465 cm^2^/V · s, due to the quite homogeneous doping contents. This result is in semiquantitative agreement with the theoretical predictions reported in ref 15, and has to be attributed to the enhancement of the carrier population in the Γ_*c*_ valley, triggered by the lowering of the Γ_*c*_-L energy barrier. As a matter of fact, despite the increased alloy scattering rates, the much lower effective mass of the Γ_*c*_ electrons, with respect to the L ones, positively impacts mobility, which is expected to exceed that of Ge when the Sn content is above 15%.^15^

**Figure 2:**
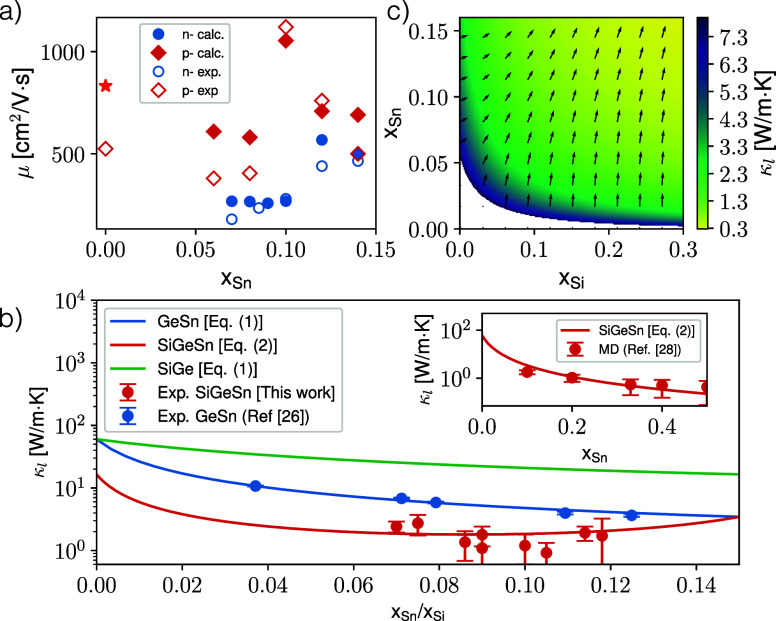
(a) GeSn electron (blue) and hole (red) mobilities as a function of Sn content, measured with Hall bar (empty symbols) and theoretically predicted by ElecTra (full symbols). For comparison, we also reported the mobility of bulk Ge at the same p-type impurity concentration of our Ge sample (red star). (b) Lattice thermal conductivity from 3-ω experiments for Ge_1–*x*_Sn_*x*_ from ref 26 (blue points) and Si_*x*_Ge_1–*x*–*y*_Sn_*y*_ with *x* + *y* = 0.15 form this work (red points). Data are shown as a function of the Sn concentration together with the estimation based on eqs 1 and 2. For comparison, the κ_l_ of SiGe (green curve) as a function of the Si concentration is also reported. In the inset molecular dynamics (MD) theoretical conductivity data (red points) from ref 28 are compared with the estimation based on eq 2. (c) Color map of the lattice thermal conductivity of Si_*x*_Ge_1–*x*–*y*_Sn_*y*_ for Si and Sn contents in [0–0.3] and [0–0.16] respectively; black arrows represent the gradient field.

Using the potential values reported in ref 15, we obtained a nice agreement with the measured mobility for the p-type materials only, while our theoretical predictions for the electron mobility overestimated the experimental data. Therefore, to calibrate the model, the alloy scattering potentials predicted for the conduction band were tuned to match the experimental data, achieving a quantitative consistency (see Figure 2a) when a 0.7 scaling factor is applied.

After this calibration procedure, we separately inspected the different channel contributions that limit the carrier mobility. In the n-type material, with up to 10% of Sn content, the alloy scattering is the dominant one, representing ∼60% of the total scattering rate and about 90% of its lattice part. At larger Sn concentrations, the relative weight of the alloy scattering decreases in favor of the electron–phonon interaction due to band-structure effects related to the energy separation between the Γ_*c*_ and L valley. For the p-type case, we find that at any composition, the alloy scattering rate is comparable to the electron–phonon one. However, in the low Sn concentration regime, their impact on the total mobility is modest, while it increases up to nearly 50% at the higher Sn concentrations.

### Lattice Thermal Conductivity of SiGeSn

Now, we focus on the assessment of the lattice thermal conductivity in a SiGeSn material system. To this aim, we have performed 3-ω experiments^38^ on a set of Si_*x*_Ge_1–*x*–*y*_Sn_*y*_ layers with *x* ∈ [0.07 – 0.12], accurately taking into account the heat propagation also through the Ge-buffer and Si-wafer material following ref 26. The investigated samples, grown on a Ge buffer in the same reactor presented above, feature a constant Ge content of 1 – *x* – *y* = 0.85 and thicknesses ranging in the 40–360 nm interval. Our estimates for κ_l_ as a function of the Sn content *y* are shown as red points in Figure 2b together with the GeSn lattice thermal conductivity measured in ref 26 for Sn contents in a similar range (blue points). As further discussed in the following, it is apparent from Figure 2b that alloying Ge with both Si and Sn induces a rapid and stronger suppression of κ_l_ with respect to the GeSn binary system. We notice that the thermal conductivity measured in the present manuscript using experimental 3-ω measurements is lower by a x3 factor than the value predicted theoretically in ref 27. by means of scattering rate formalism, while it matches well with the molecular dynamics estimation of ref 28.

Combining our 3-ω data with literature experimental^26^ and theoretical^28^ estimates, we were able through the fitting procedure detailed below to propose a phenomenological relation which describes the lattice thermal conductivity of the Si_*x*_Ge_1–*x*–*y*_Sn_*y*_ ternary system in the subset of the compositional parameter (*x*, *y*) space defined by *x* ∈ [0 – 0.3] and *y* ∈ [0 – 0.16]. The rationale underlying the choice of this parameter region is twofold. On the one side, Sn-rich alloys become thermodynamically unstable for Sn concentrations greater than ∼0.16. On the other side, TE properties of Ge-rich materials are expected to overperform with respect to their Si-rich counterpart due to the lower lattice thermal conductivity of Ge. Furthermore, we prefer not to provide TE estimates at (*x*, *y*) regions located too far from the currently available κ_l_ data.

As a starting point, we modeled κ_l_ of the Si_*x*_Ge_1–*x*_ and Ge_1–*x*_Sn_*x*_ binary alloys in terms of the thermal conductivity of their elemental constituents and of a fitting parameter *A*, relying on the following equation:^39^

$$\kappa_{\mathrm{SiGe}/\mathrm{GeSn}}\left(x\right)={\left(\frac{x}{\kappa_{\mathrm{Si}/\mathrm{Sn}}}+\frac{1-x}{\kappa_{\mathrm{Ge}}}+\frac{\left(1-x\right)x}{A_{\mathrm{SiGe}}/A_{\mathrm{GeSn}}}\right)}^{-1}$$ 1

The functional form of the above relation for κ_l_(*x*) corresponds to a U-shaped curve when *x* ∈ [0, 1]. For SiGe alloys, following ref 39, we set in eq 1 κ_Si_ = 148 W/m·K, κ_Ge_ = 60 W/m·K, and *A*_SiGe_ = 2.8 W/m·K, which corresponds to the green curve shown in Figure 2b. For the GeSn case, after fixing κ_Sn_ = 63 W/m·K, we have fitted the data reported in ref 26, obtaining *A*_GeSn_ = 0.467 W/m·K (blue curve in Figure 2b). It is apparent from Figure 2b that when alloying Ge, Sn is much more effective than Si in suppressing the lattice conductivity. Indeed, eq 1 indicates that already at 10% of Sn content, κ_GeSn_ drops at 5 W/m·K, i.e., a factor of 4 lower than the κ_SiGe_ conductivity at the same Si content.

Next, we describe the thermal conductivity of the ternary Si_*x*_Ge_1–*x*–*y*_Sn_*y*_ alloy relying on a phenomenological generalization of the above relation, defined for *x* + *y* ≤ 1:

$$\kappa_{\mathrm{SiGeSn}}(x,\;y)={\left(\frac{x}{\kappa_{\mathrm{Si}}}+\frac{y}{\kappa_{\mathrm{Sn}}}+\frac{1-x-y}{\kappa_{\mathrm{Ge}}}+\frac{x(1-x)}{A_{\mathrm{SiGe}}}+\frac{y(1-y)}{A_{\mathrm{GeSn}}}+\frac{xy}{A_{\mathrm{SiGeSn}}}\right)}^{-1}$$ 2

Eq 2 reproduces eq 1, for the binary alloys SiGe and GeSn, i.e. when *y* = 0 and *x* = 0, respectively. In the above equation, we have introduced a term proportional to the product of the Si (*x*) and Sn (*y*) concentration, controlled by *A*_SiGeSn_, which plays the role of a new fitting parameter. Our best estimate for *A*_SiGeSn_ gives 0.016 W/m·K. This value has been obtained simultaneously fitting with eq 2, the 3-ω measurements shown in Figure 2b for *x* + *y* = 0.15 and the κ_l_ theoretical estimations provided in ref 28 for *x* = *y* with *x* ∈ [0.1, 0.5], which has been obtained by molecular dynamics (MD) simulations.

The resulting κ_l_, plotted as a function of the Sn content, is shown as a red curve in Figure 2b for the *x* + *y* = 0.15 case, while comparison of eq 2 with theoretical MD points from ref 28 at *x* = *y* is reported in the inset. Besides noticing the effectiveness of the adopted fitting formula in describing κ_l_ of the ternary system in the Ge-rich domain, we stress again that the addition of Si induces, already for modest concentrations, a stronger suppression of the Ge thermal conductivity with respect to the GeSn material. As a matter of fact, typical κ_l_ values in Figure 2b are around 2 W/m·K, i.e., a factor of 2.5 lower with respect to the GeSn ones reported in ref 26 for Sn content ≈10%. κ_l_ as a function of the Si and Sn concentrations with *x* ≤ 0.3 and *y* ≤ 0.16, estimated through eq 2 is shown in Figure 2c. In the plotted domain, we observe a bowl-shaped behavior with strong gradients close to the domain boundaries, pointing approximately toward the main diagonal of the (*x*, *y*) plane, where κ_l_ reaches the lowest values, with an absolute minimum as low as 0.28 W/m·K, obtained for Si_0.3_Ge_0.54_Sn_0.16_.

## TE Performance and Discussion

Combining the fit values of the lattice thermal conductivity with the electronic properties predicted by ElecTra, we obtained the *ZT* and PF figures of merit at 300 and 400 K. Our *ZT* predictions for the p-type material at the optimal hole density are reported in Figure 3a where the black dashed curve evidence the x:y ratio equal to 3.67. This value corresponds to a Si_*x*_Ge_1–*x*–*y*_Sn_*y*_ lattice constant matching that of Ge, thus indicating the absence of biaxial deformation when a relaxed Ge buffer layer is introduced to accommodate the mismatch between the Si and SiGeSn lattice parameters. *ZT* increases at larger Sn and Si contents, since its qualitative behavior is controlled by κ_l_(*x*, *y*). As a matter of fact, different from what holds for the conduction band, alloying with Si and Ge does not induce complex band-structure effects in the valence, which maintains its single-valley character. It follows that the trend observed in Figure 3a is triggered by the suppression of κ_l_(*x*, *y*), which governs also the functional form of the hole contribution κ_h_(*x*, *y*) (Figure 3b) to the total thermal conductivity and of the optimal hole density *p*_opt_(*x*, *y*) (plotted in the SI). In the explored domain, *p*_opt_ varies in the 4–10 × 10^18^ cm^–3^ interval, which corresponds to κ_h_ values in the 0.1–0.35 W/m·K range. The lowest *p*_opt_ concentrations are expected in the Sn- and Si-rich region, where the strong suppression of κ_l_ poses an upper bound to κ_h_ when maximizing *ZT*.

**Figure 3:**
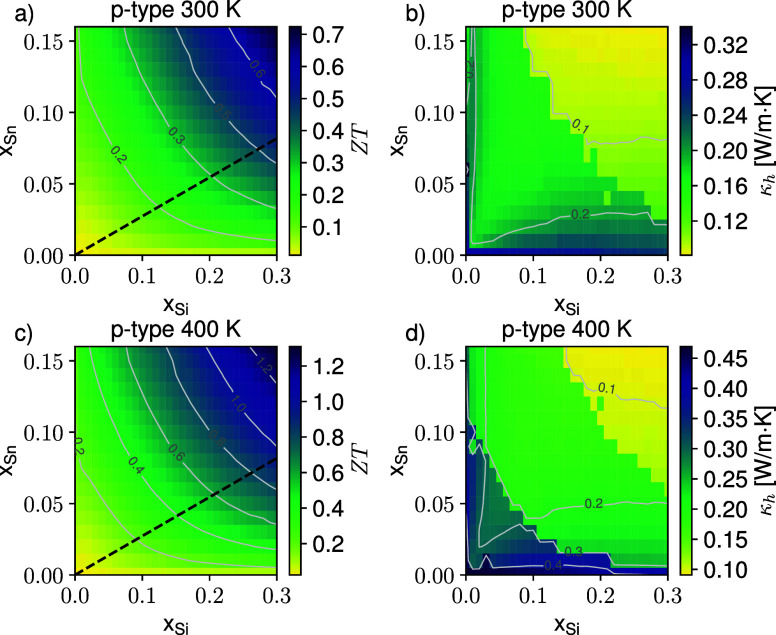
Color map of the predicted *ZT* figure of merit for p-type SiGeSn at the optimal carrier concentration at 300 K (a) and 400 K (c). The corresponding hole contribution to the thermal conductivity κ_h_ is reported in panels (b) and (d). Isolines are plotted in light gray; the black dashed curve in panels (a) and (c) evidence the lattice matching condition with the relaxed Ge buffer material.

Spanning the (*x*, *y*) parameter space, we find that *ZT* peaks for Si_0.3_Ge_0.54_Sn_0.16_ at 0.7, a value 6 times larger than the one calculated in ref 26 for the binary GeSn alloy at 0.15% Sn content. Remarkably, a modest increase of the lattice temperature triggers even larger *ZT* values as apparent from the bottom panels of Figure 3. Indeed, having in mind energy harvesting applications in the field of microelectronics leveraging on this CMOS compatible material, we have calculated TE properties also at 400 K, a typical chip operation temperature, predicting in this case *ZT* values as high as 1.3 at a hole density of about 5 × 10^18^ cm^–3^ for Sn and Si contents of 0.16 and 0.3, respectively.

We now focus on the n-type material whose predicted *ZT* figure of merit is shown in Figure 4 as a function of (*x*, *y*) in the same subset of the alloy parameter space. Top and bottom panels refer to 300 and 400 K, respectively, and as for the p-case, the right panels display the electronic contribution κ_*e*_ to the total thermal conductivity. The qualitative behavior of *ZT* differs from the one discussed above, indicating that alloy-induced band-structure effects in the conduction band play a relevant role, due to their nontrivial impact on the electronic conductivity. In particular, we expect a complex functional dependence of the effective mobility, arising from the joint action of the content-dependent alloy scattering rate and of the electron density of Γ_*c*_ carriers. As a matter of fact, as mentioned in the introduction, the conductivity mass of Γ_*c*_ electrons is much lighter than the one associated with the L valley, and the ratio between the two populations is greatly influenced by the Sn and Si content. As a result, we find that the largest *ZTs* are still found at the highest Si concentration; however, in this case, their values do not increase monotonically with the Sn content. As a matter of fact, we observe in the color maps of Figure 4a,c two regions of interest, one at high Sn content and another one at high Si and lower Sn contents. The latter is of special interest since Si has infinite miscibility in Ge, whereas Sn has limited solid-state miscibility in Si and Ge.^30^ In particular, at the *x* (Si) concentration of 0.3, we predict a peak *ZT* equal to 1.2 (1.6) at 300 K (400 K), achieved for *y* (Sn) content of 0.16, while for *y* = 0.13, we get *ZT* = 0.85 (1.5). We also notice that for the n-type material, the optimal doping densities are larger, covering the 5–7 × 10^19^ cm^–3^ range (see SI), which, however, remains experimentally accessible.^40^

**Figure 4:**
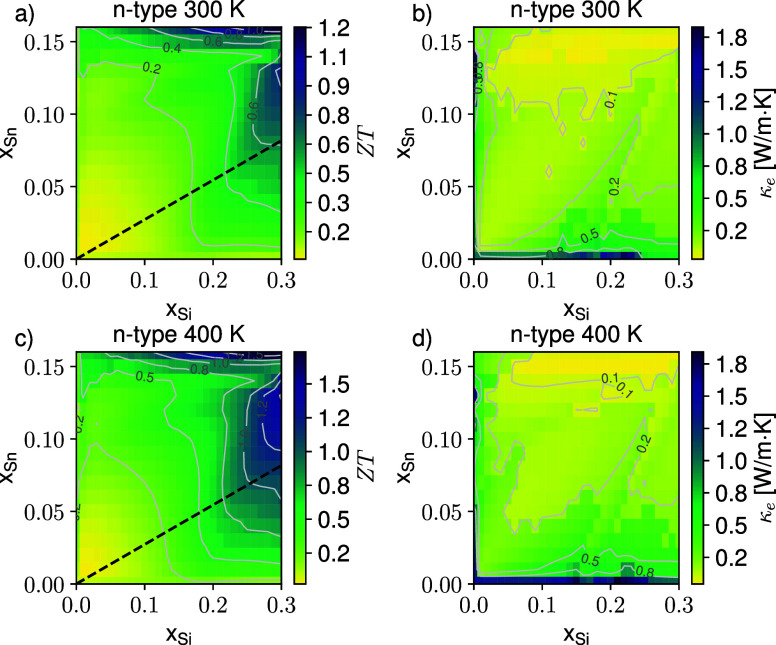
Color map of the predicted *ZT* figure of merit for n-type SiGeSn at the optimal carrier concentration at 300 K (a) and 400 K (c). The corresponding electron contribution to the thermal conductivity κ_e_ is reported in panels (b) and (d). Isolines are plotted in light gray; the black dashed curve in panels (a) and (c) evidence the lattice matching condition with the relaxed Ge buffer material.

In Figure 5, we show 300 and 400 K data for the power factor PF, another figure of merit that is of great interest for thermoelectric applications. Remarkably, the predicted PFs align with the highest values (25/30 μW/cm ·K^2^) observed in the temperature range of interest for noncompatible CMOS material systems (see refs 41–43 ). PF at this scale has also been recently measured in Si-based devices, as discussed in ref 44 where a value of 11 μW/cm ·K^2^ is reported. However, such high PF requires the nanopatterning of the active region in order to implement an electron energy filter.

**Figure 5:**
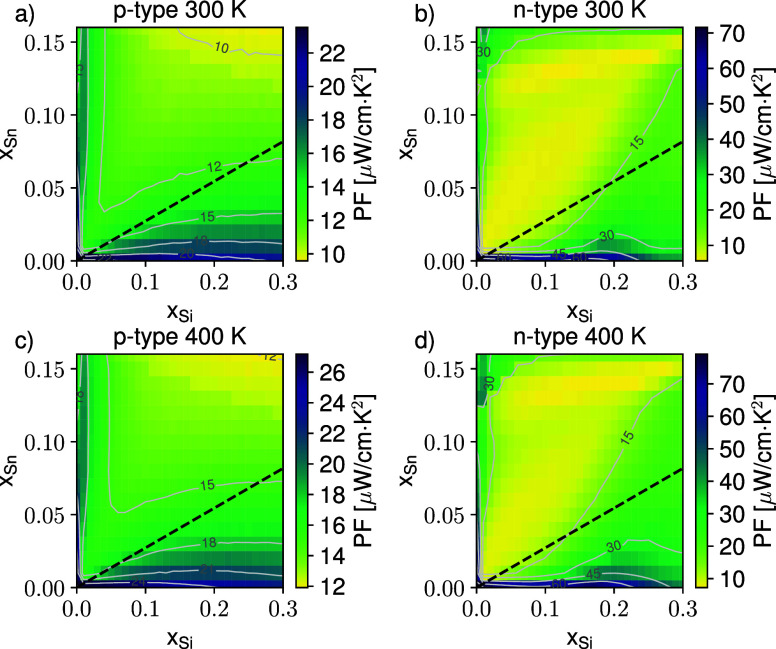
Color map of the predicted Power Factor (PF) at 300 K at the optimal carrier density for p-type (a) and n-type (b) SiGeSn alloys. Corresponding quantities calculated at 400 K are shown in panels (c) and (d). Isolines are plotted in light gray; the black dashed curve evidence the lattice matching condition with the relaxed Ge buffer material.

Finally, we focus on the special case of interest of SiGeSn alloys with a Si:Sn ratio of 3.67, since in this case the lattice parameter matches that of Ge.^45^ The predicted *ZT* and PF values for this situation are reported in Figure 6 where a monotonic behavior is observed for the hole-based systems while the n-type material exhibits a more complex behavior due to its multivalley character. This is particularly apparent in the 0.2–0.3 Si content region where the energy of the Γ valley drops down, inducing a bump in the *ZT* and a clear nonmonotonic trend in the PF.

**Figure 6:**
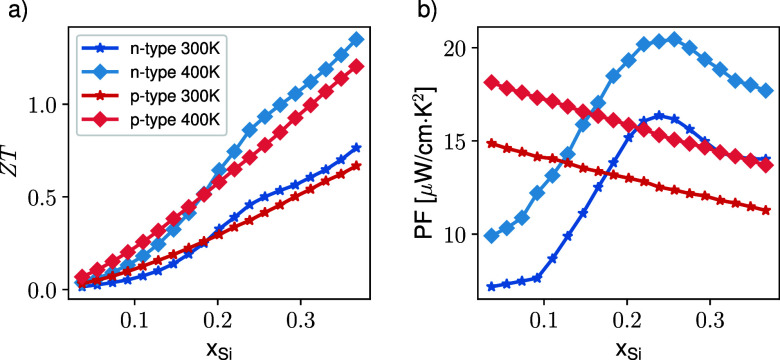
(a) Predicted *ZT* and (b) PF at the Si:Sn ratio of 3.67 as a function of the Si content at 300 and 400 K. The data are for p-type (red) and n-type (blue) alloys, as indicated in the legend.

## Conclusions

In this work, we have studied the thermoelectric properties of the novel CMOS-compatible SiGeSn ternary alloy material system. To this aim, we have combined lattice thermal conductivity 3-ω measurements, obtained from state-of-the-art SiGeSn heteroepitaxial layers deposited on Si wafers, with advanced numerical simulations of the carrier transport properties of SiGeSn alloys, performed taking into account the complex interactions stemming from its multivalley energy band landscape. The developed full stack of methods and simulation tools has allowed us to numerically explore the experimentally accessible alloy parameter space, delimited by the thermodynamic stability constraints related to the very low solubility of Sn in the Ge and Si lattices. In n-type systems, at the optimal carrier concentration, we find a *ZT* peak value at 300 (400) K of 1.2 (1.6), while the corresponding value for the p-type material is 0.7 (1.3). According to Figure 1a, it is apparent that these performances are better or on par with those of conventional TE material systems in the same temperature range, typically based on toxic/rare atomic species, which furthermore are hard or impossible to integrate in mainstream electronics. In addition, our estimations suggest that epitaxial SiGeSn is expected to overperform with respect to its polycrystal counterpart, whose TE properties have been studied in ref 29 where a room temperature peak value of *ZT* of 0.12 and 0.20 has been reported for p- and n-type systems, respectively.

On this basis, our results clearly indicate SiGeSn as a truly promising material system candidate for achieving a new CMOS-compatible TE technology, able to operate efficiently at room temperature. We therefore envisage that a transition to a SiGeSn-based TE technology could qualitatively change the application prospects of thermoelectric devices by finally allowing them to be seamlessly integrated into consumer electronics.

## Supplementary Material



## Notes

Material parameters used in the simulations and optimal carrier densities (PDF)
