# Supplementary material for: Epitaxial SiGeSn Alloys for CMOS-Compatible Thermoelectric Devices
Source: ACS Appl Energy Mater. 2025 Jun 18;8(13):9075–82. doi: 10.1021/acsaem.5c00733 (PMC12265006; doi:10.1021/acsaem.5c00733)
Supplement: Supplementary file 1 [file ae5c00733_si_001.pdf]

Supporting Information

Epitaxial SiGeSn alloys for CMOS-compatible thermoelectric devices

Patrizio Graziosi[1,†] Damiano Marian[1,‡] Andrea Tomadin,[‡] Stefano Roddaro,[¶] Omar Concepción,[§] Johnny Tiscareño-Ramírez,[§] Prateek Kaul,[§] Agnieszka Anna Corley-Wiciak,[‖,⊥] Dan Buca,[§] Giovanni Capellini,[*,‖,#] and Michele Virgilio[‡]

†CNR – ISMN, Via P. Gobetti 101, Bologna 40129, Italy

‡Dipartimento di Fisica, Università di Pisa, Largo Bruno Pontecorvo 3, Pisa 56127, Italy

¶NEST, CNR Istituto Nanoscienze, piazza San Silvestro 12, Pisa 56127, Italy

§Peter Gruenberg Institute 9 (PGI-9) and JARA-Fundamentals of Future Information Technologies, Forschungszentrum Juelich, Juelich 52428, Germany

‖IHP - Leibniz Institute for High Performance Microelectronics, Frankfurt (Oder) 15236, Germany

⊥European Synchrotron Radiation Facility 71 avenue des Martyrs, CS 40220, Grenoble Cedex 9 38043, France

#Dipartimento di Scienze, Università degli Studi Roma Tre, Viale G. Marconi 446, Roma 00146, Italy

*E-mail: giovanni.capellini@uniroma3.it

This Supplemental Material lists all the material parameters used in the simulations, in Section S1, and reports in Section S2 the optimal carrier densities, which are the values at which the ZT peak occurs.

[1] These authors contributed equally to this work

Section S1

In Table S1 we report the materials parameters used for the description of the electronic structure of the conduction band (CB). We use E_0 to indicate the valley edge, where Γ, Δ, and L indicate the valleys having the minimum at the corresponding point in the Brillouin Zone (BZ). The valley masses are indicated with *m* and are in units of electron rest mass; the 'l' and 't' indicates the longitudinal and transverse effective mass, respectively.

To keep the table light, parameters used for both CB and VB are reported only in table 4. These are: the lattice parameters *a*, the mass density ρ, and the volume of the primitive cell, $V_{primitive}$, which is used in the evaluation of the alloy scattering rate, see main text for details.

In Table S2 we list the bowing parameters for the three CB valleys and the lattice parameters, used to estimate the respective values in the alloys from the ones of the parental compounds.

Table S1, materials parameters for the CB.

**CB**	$E_{0,\Gamma}$ **eV**	$E_{0,\Delta}$ **eV**	$E_{0,L}$ **eV**	m_{Γ}	$m_{\Delta,l}$	$m_{\Delta,t}$	$m_{L,l}$	$m_{L,t}$
Si [a]	4.09	1.12	1.93	0.042	0.91	0.19	1.35	0.101
Ge [a]	0.796	0.90	0.655	0.042	1.79	0.20	1.387	0.101
Sn [b]	-0.413	0.9	0.006	0.025	1.79	0.20	1.478	0.0705

[a] M. M. Rieger and P. Vogl, *Phys. Rev. B* **48**, 14276 (1993), M. V. Fischetti and S. E. Laux, *J. Appl. Phys.* **80**, 2234 (1996).

[b] T. Brudevoll, D. S. Citrin, M. Cardona, and N. E. Christensen, *Phys. Rev. B* **48**, 8629 (1993).

Table S2, bowing parameters for the CB in separate tables for the three valleys and the lattice parameter. From reference *c*.

Γ *edge*	Si	Ge	Sn
Si	-	0.21	13.2
Ge	0.21	-	2.46
Sn	13.2	2.46	-

Δ *edge*	Si	Ge	Sn
Si	-	0.21	0
Ge	0.21	-	0.21
Sn	0	0.21	-

L edge	Si	Ge	Sn
Si	-	0	0
Ge	0	-	1.03
Sn	0	1.03	-

a	Si	Ge	Sn
Si	-	-	-
Ge	-	-	-0.026
Sn	-	-0.026	-

[c] N. S. Fernando; R. A. Carrasco; R. Hickey; J. Hart; R. Hazbun; S. Schoeche; J. N. Hilfiker; J. Kolodzey; S. Zollner *J. Vac. Sci. Technol. B* **36**, 021202 (2018).

In Table S3 we report all the scattering parameters assumed for the parental compounds Si, Ge, Sn. In the SiGeSn alloys, these values have been linearly interpolated according to the alloy composition. In Table S3, 'D' indicates the deformation potential, the subscript 'A' refers to the scattering with acoustic phonons, so-called acoustic deformation potential (ADP). We consider that the ADP deformation potential scattering process is intravalley, except between the Γ valley and the surrounding ones; indeed, when the Γ valley shifts down at increasing Sn content, it becomes close to the other valleys in the BZ, and a small momentum phonon can allow intervalley transition. The subscript 'O' indicates the intra-valley scattering with non-polar optical phonons, so-called optical nonpolar deformation potential (ODP), active only in the L valley, and the subscript 'IVS' refers to the inter-valley scattering. The valleys pair is indicated in the subscript as well. The assumed corresponding phonon energies are reported below the corresponding deformation potential in the same field.

Table S3, scattering parameters for electron transport in the CB.

CB	$D_{A,\Gamma}$ eV	$D_{A,\Delta}$ eV	$D_{A,L}$ eV	v_s m/s	$D_{o,L}$ eV/Å $\hbar\omega$, meV	$D_{IVS,\Gamma-\Delta}$ eV/Å $\hbar\omega$, meV	$D_{IVS,\Gamma-L}$ eV/Å $\hbar\omega$, meV	$D_{IVS,\Delta-\Delta}$ eV/Å $\hbar\omega$, meV	$D_{IVS,L-L}$ eV/Å $\hbar\omega$, meV	$D_{IVS,\Delta-L}$ eV/Å $\hbar\omega$, meV
Si	5 [a]	8.2 [a]	11 [a]	6.57e3	0 [c]	10 [c]<br>45	2 [c]<br>45	3.5, 0.5<br>56, 18	2 [c]<br>52	4.5 [c]<br>50
Ge	5 [a]	9 [a]	11 [a]	3.93e3	6.7 [d]<br>37	10 [d]<br>28	2 [d]<br>28	9.4, 0.78 [d]<br>37, 8.6	1.6 [d]<br>28	5.5 [d]<br>28
Sn	1.87 [b]	3.44 [b]<br><br>3.76<br>(Γ- Δ)	5.68 [b]<br><br>7.01<br>(Γ-L)	2.22e3	6.7 [e]<br>26	10<br>24	2<br>24	9.4, 0.78<br>24, 6	1.6<br>24	5.5<br>25

[a] C. Jacoboni and L. Reggiani, *Advances in Physics*, *28*(4), 493 (1979).

[b] T. Brudevoll, D. S. Citrin, M. Cardona, and N. E. Christensen, *Phys. Rev. B* **48**, 8629 (1993), M. Cardona and N. E. Christensen, *Phys. Rev. B* **35**, 6182 (1987).

[c] M. Lundstrom, Fundamentals of Carrier Transport, Cambridge University Press (2000).

[d] W. Fawcett and E. G. S. Paige *J. Phys. C: Solid State Phys.* **4** 1801 (1971).

[e] M. S. Kushwaha, *Physica B*, **101** 254 (1980), S. Wei and M. Y. Chou, *Phys. Rev. B* **50**, 2221 (1994).

We report, in table S4, the parameters used to build the band structure of the valence band (VB). Here Δ_{SO} is the spin-orbit coupling split, and the subscript LH, HH and SO refers to the light-hole, heavy-hole, split-off bands, respectively. The meaning of the other symbols is the same as in Table 1. Importantly the LH and HH effective masses for Sn were not reported in literature, so have been deduced from the Luttinger parameters as explained in the note *e*.

Finally, in Table S5 we report the scattering parameters for the parental compounds, used in the evaluation of the corresponding parameters for the alloys. The fundamental nomenclature is the same as in Table S3, with the obvious distinction that the valleys are all in Γ and are labelled as in Table S4: LH, HH, SO. A remarkable difference between the hole transport in VB and the electron transport in CB, is that in the former the ADP mechanism scatters everywhere, either intra- and inter-valley, because the valleys are all at Γ and the *k*-states belonging to each valley are close so that a nearly zero momentum phonon allows inter-valley processes. The D_O and D_{IVS} deformation potentials refers to the intra-valley and inter-valley scattering mechanisms.

Table S4, materials parameters for the VB.

**VB**	Δ_{SO} **eV**	***a*** **nm**	***ρ*** ***g/m³***	***V_{primitive}*** ***Å³***	m_{LH}	m_{HH}	m_{SO}
Si [a]	0.043	0.543	2.33	40.9	0.2	0.55	0.29
Ge [b]	0.30	0.566	5.32	47.8	0.049	0.332	0.084
Sn [c]	0.80	0.65	5.77	73.4	-0.0366 [e]	0.304 [e]	0.047 [d]

[a] C. Jacoboni, C. Canali, G. Ottaviani, A. Alberigi Quaranta, *Solid-State Electronics* **20**, 77 (1977).

[b] K. Lu Low, Y. Yang, G. Han; W. Fan; Y.-C. Yeo *J. Appl. Phys.* **112**, 103715 (2012).

[c] T. Brudevoll, D. S. Citrin, M. Cardona, and N. E. Christensen, *Phys. Rev. B* **48**, 8629 (1993).

[d] T. Brudevoll, D. S. Citrin, M. Cardona, and N. E. Christensen, *Phys. Rev. B* **48**, 8629 (1993), P. Lawaetz, *Phys. Rev. B* **4**, 3460 (1971).

[e] Estimated from the Luttinger parameters $y1$, $y2$ and $y3$; the effective mass tensor components along two directions are: $m_{100} = [1/(y1 + 2 * y2), 1/(y1 - 2 * y2)]$ and $m_{111} = [1/(y1 + 2 * y3), 1/(y1 - 2 * y3)]$. Then the effective mass is evaluated as $m_{eff} = 2./(1./m_{100} + 1./m_{111})$. The linear combination these for GeSn in the x_{Sn} < 0.2 range fits at the second decimal with the values reported in the note *b* above and *e1* below, for the LH and HH valleys, and with the value reported in *e2* for the split-off band.

[e1] S. Q. Liu; S.-T. Yen, *J. Appl. Phys.* **125**, 245701 (2019)

[e2] N. S. Fernando; R. A. Carrasco; R. Hickey; J. Hart; R. Hazbun; S. Schoeche; J. N. Hilfiker; J. Kolodzey; S. Zollner *J. Vac. Sci. Technol. B* **36**, 021202 (2018).

Table S5, scattering parameters for hole transport in the VB.

VB	v_s m/s	$D_{A,LH}$ eV	$D_{A,HH}$ eV	$D_{A,SO}$ eV	$D_{A,LH-HH}$ eV	$D_{A,LH-SO}$ eV	$D_{A,HH-SO}$ eV	D_o eV/Å hw,meV	D_{IVS} eV/Å hw,meV
Si	6.57e3	6.6 [a]	6.6 [a]	6.6 [a]	6.6 [a]	6.6 [a]	6.6 [a]	10.45 [b]<br>62 [b]	10.45 [b]<br>62 [b]
Ge	3.93e3	5 [b]	5 [b]	5 [b]	5 [b]	5 [b]	5 [b]	9 [b]<br>38 [c]	9 [b]<br>38 [c]
Sn	2.22e3	1.87 [d]	8.9 [d]	5.54 [d]	1.14 [d]	5.54 [d]	5.54 [d]	9<br>25 [e]	9<br>25 [e]

[a] Z. Li, P. Graziosi, and N. Neophytou, *Phys. Rev. B* **104**, 195201 (2021), J. Kim and M. V. Fischetti, *J. Appl. Phys.* **108**, 013710 (2010).

[b] C. Jacoboni and L. Reggiani, *Advances in Physics*, *28*(4), 493 (1979).

[c] S. Wei and M. Y. Chou, *Phys. Rev. B* **50**, 2221 (1994).

[d] T. Brudevoll, D. S. Citrin, M. Cardona, and N. E. Christensen, *Phys. Rev. B* **48**, 8629 (1993).

[e] M.S. Kushwaha, *Physica B*, **101** 254 (1980).

Section S2

In this section we report the color maps for the optimal carrier densities, i.e. the values at which the ZT peaks as a function of the Sn and Si concentrations, calculated at 300 and 400 K. These values have been discussed in the main text.

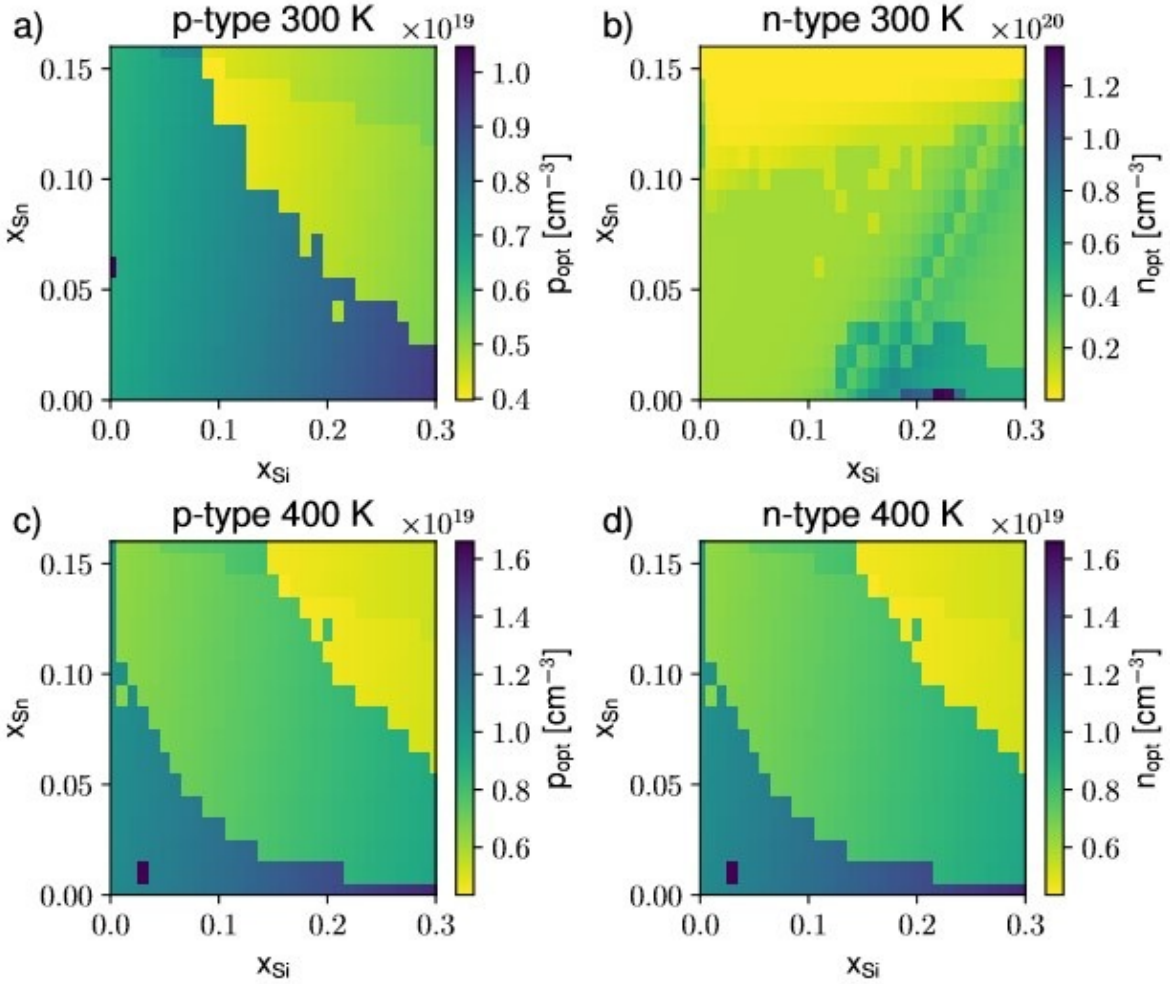

Figure S1: Optimal carrier densities at 300 and 400 K
